# Does Pedestrian Danger Mediate the Relationship between Local Walkability and Active Travel to Work?

**DOI:** 10.3389/fpubh.2016.00089

**Published:** 2016-05-09

**Authors:** Sandy J. Slater, Lisa Nicholson, Haytham Abu Zayd, Jamie Friedman Chriqui

**Affiliations:** ^1^Division of Health Policy and Administration, Institute for Health Research and Policy, School of Public Health, University of Illinois at Chicago, Chicago, IL, USA; ^2^Institute for Health Research and Policy, Chicago, IL, USA

**Keywords:** physical activity, active travel, built environment, walkability, pedestrian danger

## Abstract

**Background:**

Environmental and policy factors play an important role in influencing people’s lifestyles, physical activity (PA), and risks for developing obesity. Research suggests that more walkable communities are needed to sustain lifelong PA behavior, but there is a need to determine what local built environment features facilitate making being active the easy choice.

**Purpose:**

This county-level study examined the association between local walkability (walkability and traffic calming scales), pedestrian danger, and the percent of adults who used active transport to work.

**Methods:**

Built environment and PA outcome measures were constructed for the 496 most populous counties representing 74% of the U.S. population. Geographic information system-based walkability scales were constructed and include a census of roads located within the counties using 2011 Navteq data. The pedestrian danger index (PDI) includes data collected from the Fatality Analysis Reporting System 2009–2011, and measures the likelihood of a pedestrian being hit and killed by a vehicle. Four continuous outcome measures were constructed using 2009–2013 American Community Survey county-level 5-year estimates. The measures represent the percentage of workers living in a county who worked away from home and (1) walked to work; (2) biked to work; (3) took public transit; and (4) used any form of active transport. Linear regression and mediation analyses were conducted to examine the association between walkability, PDI, and active transport. Models accounted for clustering within state with robust SEs, and controlled for median household income, families with children in poverty, race, ethnicity, urbanicity, and region.

**Results:**

The walkability scale was significantly negatively associated with the PDI (β = −0.06, 95% CI = −0.111, −0.002). In all models, the PDI was significantly negatively associated with all active travel-related outcomes at the *p* < 0.01 level. The walkability scale was positively associated with all four outcomes at the *p* < 0.01 level. Results showed that the significant positive relationship between local walkability and the four active transport outcome measures was partially mediated by the PDI. We found no association between traffic calming, the PDI, and the active transport outcomes.

**Conclusion:**

Results from this study show that, at the county-level, walkability is associated with active travel, and this association is partially mediated by an index of pedestrian safety.

## Introduction

The *Physical Activity Guidelines for Americans* (1) recommend that adults get at least 150 min per week of moderate intensity or 75 min per week of vigorous-intensity physical activity (VPA) through aerobic exercise, including brisk walking/biking. However, most Americans do not get the recommended levels of physical activity (PA) (2). In fact, most Americans get well below the recommended levels of PA, with less than half of adults self-reporting that they meet national recommendations (3, 4), and <5% of adults whose PA has been objectively measured through accelerometer meet the recommendations (2). Therefore, increasing population-level participation in PA has been identified as a public health priority by the Centers for Disease Control and Prevention (5). Thus, research is needed to identify what factors influence active travel choice, as well as which factors may facilitate or act as barriers to active travel.

Walking is the most prevalent form of exercise in adults (6, 7). Recognizing that walking is one PA strategy that can be achieved for leisure, exercise, and active travel (i.e., walking, biking, or taking public transit to work), the Surgeon General’s recently announced Step it Up! initiative will promote and support walking and walkable communities (8). Active travel has been shown to provide up to 44.3 min weekly of moderate intensity PA for adults in neighborhoods identified as having high walkability streets in comparison to only 12.8 weekly minutes of moderate intensity PA in neighborhoods identified as having low walkable streets (9). Adults who use active travel modes to go to and from work also have higher levels of daily PA than those who do not (10). Furthermore, a recent literature review shows that adults can achieve anywhere from 8 to 33 additional minutes of walking daily if they take public transit to and from work (11). Yet, few people actually use active travel to get to work. Currently, the percentage of adults reporting walking or biking to work averaged 3.4% across 2008–2012; younger workers, i.e., those aged 16–24, averaged 6.8% (12). Community walkability, as well as walking and PA more broadly, vary greatly based on where people live (13–16) with overall rates of walking and PA low nationwide. A number of governmental, quasi-governmental, and authoritative bodies have stated that policy and environmental strategies are critical to population-wide prevention of obesity and increased healthful behaviors, including PA and walking (17–25).

Environmental and policy factors play an important role in influencing people’s lifestyles, PA, and risks for developing obesity [(19, 26), p. 320–332; (27–31)]. Evidence shows that community- and street-scale urban design promotes PA (19). Characteristics of communities that facilitate PA include more compact developments with a mix of residential, commercial, retail, and recreational destinations; traditional neighborhood design that provides street and sidewalk connectivity; transportation infrastructure; and proximity to recreational areas/facilities (19).

By contrast, sprawling communities requiring the use of automobiles and communities with limited transportation infrastructure, poor street/sidewalk connectivity, lack of sidewalks or bike paths, and high traffic volume provide unsafe walking environments and have lower rates of active transportation or PA (13, 32). Research suggests that improving pedestrian and cyclist safety through the creation of more walkable communities can lead to increases in active transportation.

Infrastructure changes that create more walkable neighborhoods have been shown to improve pedestrian safety by reducing pedestrian injuries caused by motor vehicles (33). Traffic calming measures, which affect the speed and volume of car traffic on roads, have also been shown to reduce the number of traffic accidents in neighborhoods up to 15% (34, 35). Furthermore, traffic calming strategies have been identified as an effective method to increase walking and improve overall health (34, 36, 37). Yet, a review by Rothman et al. (37) on how specific features of the built environment relate to both walking in elementary school children and child pedestrian injury also found mixed results with some street features associated with increased injuries, such as sidewalks and street parking. There are also a handful of studies that have found an association between low traffic roads, i.e., those with some traffic calming, and increased bicycling as a mode of transportation (38). However, these studies focus on one or a handful of communities. To our knowledge, no studies have examined the association between traffic calming measures and active travel or pedestrian danger at the national level. Furthermore, as summarized in this background section, existing evidence examines the association between traffic calming or walkability measures and active travel or pedestrian danger. No previous study has examined the association between local walkability, pedestrian danger, and the percent of adults who used some mode of active travel at the county level. Given these evidence gaps, and in order to inform policy change, more research is needed to determine what is the most effective infrastructure to not only encourage active travel to work as a means to increase overall PA, but also ensure that it can be done safely.

Ding and Gebel (39) also state the need for future research to examine potential mediators of the built environment and PA association. Mediation analyses can help provide insights into some of the mechanisms that either encourage or discourage active travel behavior by allowing for the consideration of how a third variable affects the relation between two other variables. Specifically, this study builds on previous evidence gaps by employing mediation analyses to examine whether a walkability scale and a traffic calming scale indirectly work through pedestrian danger to increase active travel rates in a national sample of counties. Our primary aim was to test the hypothesis that a county-level walkability scale will be associated with lower scores of the pedestrian danger index (PDI), which in turn will be associated with higher prevalence of active travel.

## Materials and Methods

### Sample

This was a cross-sectional study conducted between May 2012 and December 2015. Built environment and PA outcome measures were constructed for the 496 most populous counties representing 74% of the U.S. population. Counties are located in all states with the exception of Wyoming (WY). Geographical information system (GIS)-based walkability and traffic calming scales were constructed and include a census of roads located within the counties using 2011 Navteq data. The University of Illinois at Chicago (UIC) Institutional Research Board deemed that this study did “not involve human subjects” (research protocol #2011-0880).

### Data Sources and Measures

#### American Community Survey

County-level outcomes and contextual controls were obtained from the Census Bureau’s American Community Survey (ACS) 2009–2013 5-year estimates (40). The ACS is an annual survey that helps communities to plan investments and services and includes socio-demographic characteristics for each community. While the ACS 1-year estimates provide the most current data, they are limited to jurisdictions with populations >65,000 persons. The 3-year estimates provide more precise estimates but are limited to jurisdictions with >20,000 persons. For this study, we used the 5-year estimates because they include all jurisdictions nationwide, which was necessary because the policy measures are based on all jurisdictions within each county that represented >0.5% of the given county population, including very small jurisdictions that are not captured in the 1- and 3-year data files. The 5-year estimates represent the most precise estimates (40). ACS data were used to construct the four active travel-related outcome measures described below and were linked to all other data sources using county-level geocodes.

ACS respondents were asked, “How did this person usually get to work LAST WEEK?” Response categories included car, truck, or van; bus or trolley bus; streetcar or trolley car; subway or elevated railroad; ferryboat; taxicab; motorcycle; bicycle; walked; and worked at home. Using these response categories, four continuous outcome measures were constructed using 2009–2013 ACS county-level 5-year estimates. The first measure represents the percentage of workers living in a county who worked away from home and walked to work. The second measure represents the percentage of workers living in a county who worked away from home and took public transit. The third measure represents the percentage of workers living in a county who worked away from home and rode their bike to work. The fourth measure represents the percentage of workers living in a county who worked away from home and used active transport (includes walking, biking, or using public transit) to get to work. County-level demographics included: percentages of families with children living in poverty, % non-Hispanic White, % non-Hispanic Black, % Hispanic, median household income, region of the U.S. The percentage of land area in a county that was urban was generated from 2010 Census data.

#### NAVTEQ

ArcGIS 9.1 software was used to access NAVTEQ 2011 data with third quarter updates. NAVTEQ data provided information for each county regarding the number of four-way street-level intersections and a count of all street level intersections. These measures were used in combination with other density measures to create two scales: walkability and traffic calming. The scales comprised a combination of both macro- and micro-scale walkability features. For each scale, items are first standardized then a summated standardized scale is constructed. The final scale values are also adjusted by a factor of +1 to account for negative and zero values. The traffic calming scale, described in detail elsewhere (41), accounts for the level of safety of the local street network. Briefly, in order to create the single outcome measure of traffic calming, confirmatory factor analysis (CFA), a specific type of structural equation model (SEM) was used to identify the factors associated with the latent construct of traffic calming. All models tested included one latent variable and a series of observed items with and without correlated errors. Chi-square difference tests were used to test if one model was a significant improvement over previous models. All fit statistics available in Stata 12 (chi-square, RMSEA, TL, SRMR, and CD) were also evaluated in deciding on the best model fit for the data. The best model fit included a five-factor model (intersection density, low mobility streets, roundabouts, dividers, and parking). The walkability scale draws upon a similar measure created by Slater and colleagues (42), which was adapted from the scale created and updated by Ewing and colleagues (43). The scale was calculated using a combination of NAVTEQ 2011 data and ACS 2007–2011 data and includes the following measures: percentage of four-way intersections (NAVTEQ), intersection density (NAVTEQ), housing unit density (ACS), and population density (ACS) per square mile. A factor was extracted using principal components analysis to represent the level of compactness, i.e., high density areas of street networks and population, or walkability, of a county.

#### Fatality Analysis Reporting System

National Highway Traffic Safety Administration’s Fatality Analysis Reporting System (FARS) data for 2009–2013 were used to construct the Pedestrian Danger Index described below. FARS contains a census of fatal crashes within the 50 states, the District of Columbia, and Puerto Rico. FARS data have been collected since 1975 and contain over 100 different data elements that characterize the crash, vehicle, and people involved. Included in the data is information on accidents involving pedestrians and bicyclists, as well as information on the time and location of the crash. NHTSA also has information on the exact latitude and longitude of the accident. Data were matched to the study counties using county and state FIPS codes provided by FARS.

The PDI was constructed using existing methods developed by Smart Growth America and the National Complete Streets Coalition (44) and includes data collected from the 2009–2013 FARS databases. The PDI allows for comparisons at the national level, of pedestrian safety across different areas. The PDI uses as its denominator the percent, or share, of local commuters who walk to work; it provides a measure of how many people are likely to be out walking to work each day.

The PDI was constructed, based on the documentation and explanation provided in the Dangerous by Design Report (44), by taking the county-level counts of pedestrian deaths extracted from FARS and dividing it by the Census county-level population per year. This number was then multiplied by 100,000 to create a measure of the county-level pedestrian fatality rate per 100,000 persons. We then averaged the fatality rate across the 5 years of data and then divided it by the percent of people in the county who walk to work.

### Analysis

**Figure 1:**
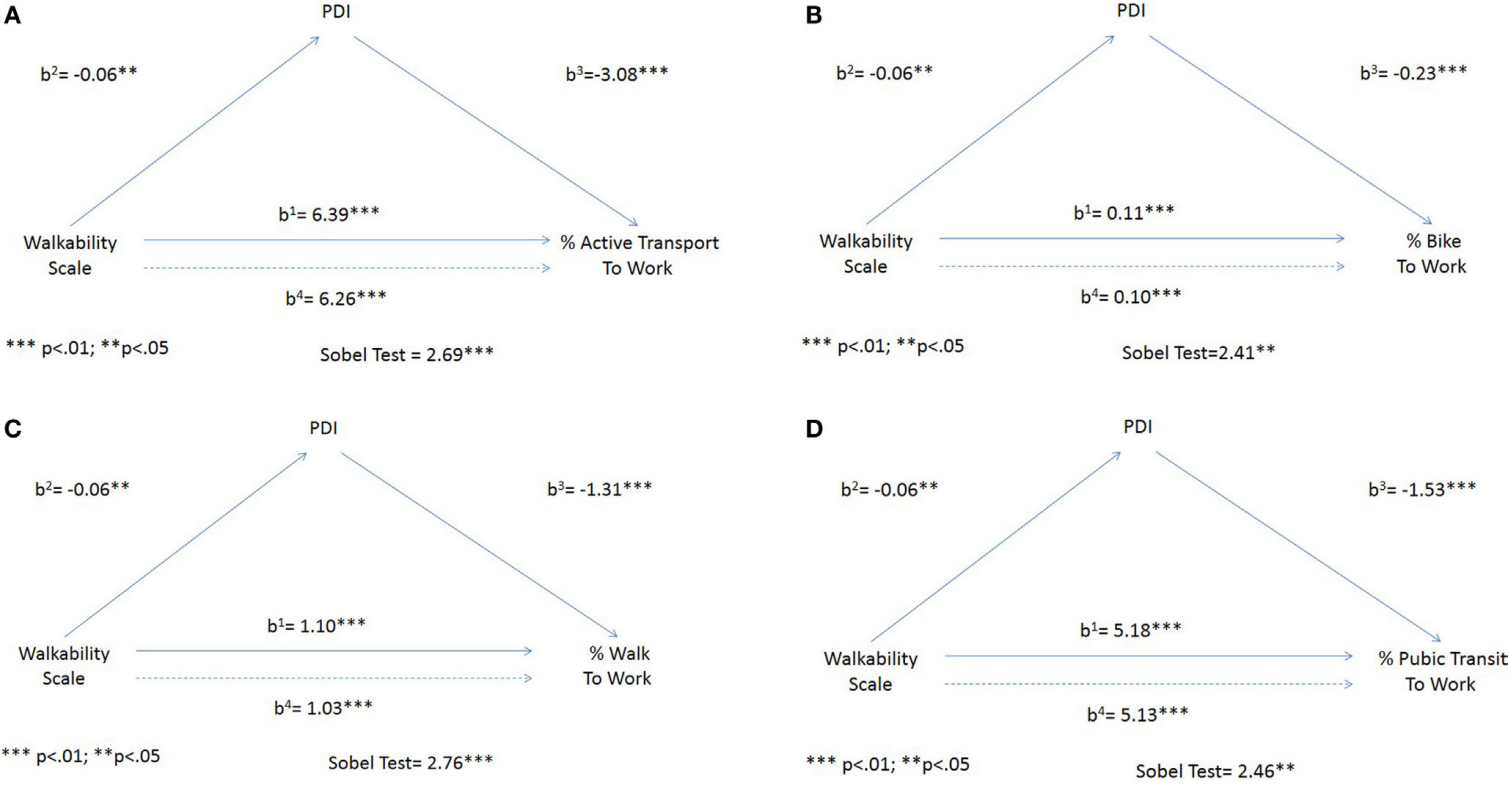
**Mediation analysis path diagrams with separate regression coefficients (b^1^, b^2^, b^3^, and b^4^) for each regression model for the full sample of counties (*n* = 496)**. **(A)** % active transport to work, **(B)** % bike to work, **(C)** % walk to work, and **(D)** % public transit to work.

Descriptive statistics (means, SDs, minimum, and maximum values) were examined and are presented in Table 1. Due to the clustered nature of the data, all linear regression models were clustered within states with robust SEs. Data were prepared and analyzed using the STATA SE v. 13.1 and used two-sided tests with significance levels of 0.10, 0.05, and 0.01. All models included the following county-level covariates: median household income, percentage Non-Hispanic Whites, percentage Non-Hispanic Blacks, percentage Hispanics, percentage of families with children living in poverty, percentage of the county that was an urban area and dummy variables for regions of the U.S. [West, Midwest, South, and Northeast (omitted reference)]. Levels of significance were indicated by asterisks and noted at the bottom of the regression table (see Table 2 for full models, including covariates, independent variables, and mediator variable). See Figure 1 for path model coefficients and Sobel test statistics as described below.

**Table 1:** **Descriptive statistics of the sample of counties (*N* = 496)**.

	Mean	SD	Min	Max
**Outcome measures**				
% Active transport	6.57	8.82	0.60	86.78
% Walk to work	2.64	2.00	0.30	22.42
% Bike to work	0.58	0.84	0.00	9.64
% Public transit to work	3.35	7.13	0.07	63.67
**Independent variables**				
Walkability scale	1.00	1.00	0.72	17.99
Traffic calm scale	1.00	1.00	0.42	10.54
**Mediator variables**				
Pedestrian danger index (PDI)	0.92	0.76	0.00	3.73
**Covariates**				
Median household income	56673.47	14460.53	29806.00	122238.00
% Non-hispanic white	67.97	19.08	3.57	95.65
% Non-hispanic black	11.84	12.56	0.27	69.45
% Hispanic	13.27	14.59	0.87	95.50
% Families w/kids in poverty	8.21	3.59	1.75	25.24
West	0.18	0.39		
Midwest	0.22	0.42		
South	0.39	0.49		
Northeast^a^	0.20	0.40		
% Urban	82.22	14.66	31.86	100

*^a^Omitted region*.

**Table 2:** **Linear regression models examining walkability, traffic calming, and PDI and on all outcomes**.

	Walkability Scale Full Models (*N* **=** 496)				Traffic Calming Full Models (*N* **=** 496)			
	% Active transport	% Walk	% Bike	% Public transit	% Active transport	% Walk	% Bike	% Public transit
PDI	−2.29*** (0.35)	−1.18*** (0.11)	−0.22*** (0.04)	−0.89*** (0.25)	−3.10*** (0.51)	−1.31*** (0.13)	−0.23*** (0.05)	−1.55*** (0.38)
Walkability Scale	6.26*** (1.32)	1.03*** (0.10)	0.10*** (0.04)	5.13*** (1.35)				
Traffic Calm Scale					0.38 (0.36)	0.02 (0.06)	−0.03 (0.04)	0.38 (0.29)
Med HH Income	−0.25 (0.31)	−0.45*** (0.08)	−0.17*** (0.05)	0.37 (0.27)	0.14 (0.52)	−0.23*** (0.05)	−0.39*** (0.10)	0.70 (0.44)
% Non-Hisp White	−0.19*** (0.05)	−0.05*** (0.01)	−0.01* (0.01)	−0.13*** (0.05)	−0.33*** (0.10)	−0.08*** (0.02)	−0.02* (0.01)	−0.24*** (0.09)
% Non-Hisp Black	−0.06 (0.06)	−0.03** (0.01)	−0.01 (0.01)	−0.02 (0.04)	−0.14 (0.10)	−0.05** (0.02)	−0.01 (0.01)	−0.08 (0.08)
% Hispanic	−0.13*** (0.05)	−0.05*** (0.01)	−0.01* (0.01)	−0.07* (0.04)	−0.24*** (0.09)	−0.07*** (0.02)	−0.02** (0.01)	−0.16** (0.07)
% Families w/Kids Poverty	−0.21 (0.14)	−0.08** (0.04)	−0.06*** (0.02)	−0.07 (0.12)	0.07 (0.26)	−0.04 (0.05)	−0.06** (0.03)	0.16 (0.23)
West	−1.62* (0.97)	−0.38* (0.22)	0.93*** (0.16)	−2.17** (0.86)	−6.69*** (1.94)	−1.20*** (0.36)	0.86*** (0.17)	−6.35*** (1.68)
Midwest	−3.18*** (0.63)	−0.96*** (0.20)	0.10 (0.07)	−2.32*** (0.51)	−6.19*** (1.29)	−1.46*** (0.28)	0.05 (0.07)	−4.79*** (1.08)
South	−3.48*** (0.81)	−0.81*** (0.19)	0.22*** (0.08)	−2.89*** (0.71)	−6.78*** (1.60)	−1.37*** (0.27)	0.16** (0.08)	−5.57*** (1.40)
% Urban	0.02 (0.01)	−0.01** (0.00)	0.01*** (0.00)	0.02* (0.01)	0.08*** (0.02)	0.00 (0.00)	0.01*** (0.00)	0.07*** (0.01)

***p < 0.01.

**p < 0.05.

*p < 0.10.

*Unstandardized coefficients with SEs in parentheses*.

Single mediation regression models were used to examine the effectiveness of two scales (walkability and traffic calming) with and without the presence of PDI and to help determine if the effects of walkability and traffic calming components were reduced in counties with high PDI. We employed two major approaches to statistical mediation analysis: (a) causal steps (45) and (b) the Sobel test (46). All individual outcomes were examined separately and since the walkability and traffic calming scales share one component (intersection density), these measures were also examined in separate models only. The following equations apply to all approaches (47), where Y is the dependent variable, X is the independent variable, M is the mediator, *c* is the coefficient relating the independent and dependent variables, *c*’ is the coefficient relating the independent and dependent variables adjusting for the mediator, and *b* is the coefficient relating the mediator to the dependent variable adjusted for the independent variable. Intercepts are displayed as (*i*_1_, *i*_2_, *i*_3_) and (*e*_1_, *e*_2_, *e*_3_) are residuals.

(1) $$\text{Y}=i_{\text{1}}+c\text{X}+e_{\text{1}}$$

(2) $$\text{M}=i_{\text{3}}+a\text{X}+e_{\text{3}}$$

(3) $$\text{Y}=i_{\text{2}}+c’\text{X}+b\text{M}+e_{\text{2}}$$

We first followed Baron and Kenny’s classic work of causal step analysis, which determined significant mediation when four conditions were met. Using our data (see Figure 1), the walkability scale needed to be significantly associated with the mediator PDI. Second, the PDI mediator variable needed to be significantly associated with the outcome. Third, the walkability scale needed to be significantly associated with the outcome. Lastly, the coefficient relating the walkability scale to the active transport to work outcomes must be larger (in absolute value) than the coefficient relating the walkability scale to the outcome in the regression model with both the walkability scale and the mediator variable predicting active transport. In our analysis, the walkability scale met all four criteria for mediation according to the Baron and Kenny causal steps approach and, therefore, additional mediation approaches were analyzed. Since the traffic calming scale did not meet the criteria, no additional analyses were warranted and only the full regression models as per Eq. 3 above were presented.

Significance of partial mediation of the relationship between the walkability scale and the active transport outcomes were then evaluated using the Sobel test (46). The Sobel test assesses whether the indirect effect of the independent variable of interest via the mediator is significantly different from 0. All Sobel test statistics are presented in Figure 1.

## Results

### Sample Characteristics

Table 1 presents descriptive statistics for the sample of counties. On average 6.57% (range: 0.60–86.78) of workers living in a county used some form of active transport to get to work. The most prevalent mode was public transit to work (3.35%, range: 0.07–63.67), followed by walking (2.64%, range: 0.30–22.42), and finally biking to work (0.58%, range: 0–9.64). The mean PDI was 0.92 (range: 0–3.73), which is an indication of the likelihood of a person outside of the vehicle being killed by a vehicle. For example, in our sample the average annual pedestrian fatality rate was 1.66 (S.D. 0.91, range: 0–6.84) per 100,000 (2009–2013 average). The average PDI represents an average fatality rate of approximately 2.2 pedestrian fatalities per 100,000 people. The standardized walkability and traffic calming scales both had means of one. To put this into context, an example of a county with the average walkability scale of 1 is Orange County, FL, USA, which includes the city of Orlando, has a county-level population density of 3,860 and intersection density of 57 per square mile. An example of a county with the average traffic calming scale of 1 is Middlesex County, NJ, USA, which houses Edison, NJ, USA, the fifth largest municipality in the state. Middlesex County has an intersection density of 53 per square mile, and county-level counts of 2,787 low mobility streets, 9 roundabouts, 737 dividers, and no on-street parking. In general, most counties across the country had low levels of walkability and traffic calming infrastructure. Finally, the mean percentage of urban land areas located within the sample of counties was 82.22%.

### Results of Mediation Analyses

#### Walkability Scale Results

Results of the multivariate linear regression models are presented in Table 2. The walkability scale was significantly negatively associated with the PDI (β = −0.06, 95% CI = −0.111, −0.002). In all eight models, the PDI was significantly negatively associated with all active travel-related outcomes at the *p* < 0.01 level. The walkability scale was positively associated with all four outcome measures at the *p* < 0.01 level. A one SD change in the walkability scale, changing from a mean of 1 to a mean of 2, would result in the average percent of people who walk to work changing from an average of 2.64–3.67% at the county level, or a 28% relative increase in the prevalence of walking to work. To put this in context, Essex County, NJ, USA, which includes Newark, has a walkability scale score that is one SD above the mean, and St. Louis County, MN, USA, which includes Duluth, has the minimum walkability scale score in our sample (which is approximately a 0.5 SD below the mean).

Mediation analysis path diagrams can be found in Figure 1. The b^1^ path shows the direct association between the walkability scale and the likelihood that workers will use active transport to get to work. The b^2^ path shows the direct association between the local walkability scale and the PDI (mediator). The b^3^ path shows the direct association between the PDI and likelihood that workers will use active transport to get to work. The b^4^ path shows the association between the walkability scale and the likelihood that workers will use active transport to get to work after accounting for the PDI. The figures show that PDI partially mediates the association between local walkability and the four active travel-related outcomes. Results showed that 3% of the significant positive relationship between local walkability and workers who used active transport to work was partially mediated (Sobel test statistic 0.24, *p* = 0.007) by the PDI. For workers who walked to work, results showed partial mediation of 9% between local walkability and the outcome (Sobel test statistic 0.11, *p* = 0.006). We found that 22.5% of the significant positive relationship between local walkability and workers who biked to work was partially mediated (Sobel test 0.02, *p* = 0.015) by the PDI. Finally, results showed that 1.8% of the significant positive relationship between local walkability and workers who took public transit was partially mediated (Sobel test 0.11, *p* = 0.014) by the PDI.

#### Traffic Calming Analyses

We found no association between traffic calming and the PDI or the four outcome measures. Sensitivity analyses showed that the traffic calming scale was positively (β = 0.77, 95% CI = 0.080, 1.46) associated with the percent of workers who took public transit and biked to work (β = 0.10 95% CI = 0.04, 0.17) (results not shown), but results were insignificant once region and urbanicity were added to the models. No mediating relationships were found between traffic calming, PDI, and the four outcome measures.

## Discussion

### Walkability Scale

The results of the analyses provide evidence supporting our primary study hypothesis that counties scoring higher on the walkability scale had better pedestrian safety, i.e., lower PDI scores, than counties scoring lower on the walkability scale. These counties, in turn, had higher prevalence of adult active travel across multiple modes – walking, biking, and public transit use; and there was a significant mediation effect – the walkability scale worked through the PDI and was associated with higher prevalence of active travel to work.

Across all study models, we found that counties with higher PDI scores, i.e., a higher likelihood of a pedestrian being killed by a vehicle, was adversely associated with any form of using active transport to get to work. Consistent with previous research (48), the walkability scale was positively associated with all four active travel-related outcomes, including bicycling. Results of the models also showed that counties with higher scores on the walkability scale were associated with lower PDI scores. This scale can serve as a proxy for certain micro-scale street features. For example, in previous sensitivity analyses conducted by the study team using an identical GIS-constructed walkability scale as the one used in this study, and a walkability scale constructed using data collected from on-the-ground street segment audits, we found high correlation between some of the micro- and macro-scale measures. First, the proportion of streets in a community with sidewalks is highly correlated with intersection density (*r* = 0.80, *p* < 0.0001) and, second, the proportion of streets in a community with mixed land use is highly correlated with both housing and residential density (*r* = 0.73 and 0.68, *p* < 0.0001, respectively). Sidewalk infrastructure, in particular, provides a natural barrier between pedestrians and cars, which would lead to fewer pedestrian fatalities with motor vehicles. While the walkability scale included in this analysis can serve as a proxy for some micro-scale street features, there are other potentially important measures that it does not capture, e.g., presence of crosswalks, pedestrian signals at traffic lights, and biking infrastructure, such as bike lanes and bike parking. Future research should examine more refined measures of micro-scale street features in similar analyses. Yet, overall these findings provide some evidence supporting the need to enact and implement complete streets or other active living-oriented zoning policies, i.e., policies that create streets that are safe and convenient for all users.

Consistent with previous research (48), study findings suggest that more walkable streets are associated with higher prevalence of biking to work. By contrast, a literature review conducted by Reynolds et al. (49) also found that bicyclists who rode on sidewalks had a higher likelihood of injury, anywhere from 1.8 to 16 times greater than cyclists who rode on the street. It is possible that counties with better walking infrastructure may also have improved biking infrastructure, such as designated bike lanes on streets, or off-road bike paths. To our knowledge, no previous study has examined the prevalence of both the walking and biking built environment infrastructure and its association with walking and biking behavior. Future research should examine these environments and behaviors simultaneously.

### Traffic Calming Scale

Although most of our results examining the association between the county-level traffic calming scale and adult active travel were null, this study also adds to the very limited literature examining the relationship between traffic calming and adult active travel. Much of the U.S. literature examining traffic calming features has focused on youth active travel to school. These studies consistently show that traffic calming is associated with more youth active travel to school and less child pedestrian injury (37). By contrast, in our final models, we found no evidence that higher scores of the county-level traffic calming scale were associated with reduced PDI scores and as previously stated, only found positive associations between traffic calming and adult active travel-related outcomes in models that did not control for region or urbanicity. Literature examining traffic calming features and adult active travel in the U.S. is sparse. It is possible that traffic calming would affect youth and adult active travel differently. Traffic calming addresses cars moving on the street only, whereas measures of walkability affect the pedestrian environment and create safer places to walk that are separate from car traffic. Parents may be more sensitive to traffic volume, and how it may affect their child walking to and from school regardless of the level of walkability of the streets. Adults may not be as sensitive to traffic volume when walking. Traffic calming features may also be more targeted in their placement near schools, which tend to be located in or near residential areas, or other targeted areas in a community with a small radius of benefit rather than dispersed more broadly throughout a community. Although our outcome measures are specific to active travel, they do not necessarily match well with the locations of traffic calming features in our sample of counties. Future research should examine adult active travel behavior that occurs near the exact location of traffic calming features.

Nicholson et al. (41) found similar results in a study that also examined traffic calming across a national sample of communities. They found that certain traffic calming features were more prevalent in the West, and less densely populated areas had few, if any, traffic calming features. Walkability features are much more prevalent and uniformly dispersed across communities. For example, most locations have sidewalks, traffic lights, and cross walks on some local streets (50). However, the installation of traffic calming features has spread less evenly across the country (50). This may help explain why previous research (38), which was conducted in a densely populated city located in the West, found an association between traffic calming and bicycling for transportation and we did not. These previous studies (38, 51) that found lower traffic volume, or slower speed streets, which are the result of traffic calming, were both positively associated with bicycling frequency. Future research is needed to determine if traffic calming can be effective in less densely populated areas and other regions of the country.

### Limitations and Conclusion

Major strengths of this study include the use of objective walkability, traffic calming and PDI measures, and the use of a national sample of counties. It is also one of the first studies to examine the mediating, or indirect effect of PDI on the relationship between local walkability and traffic calming scales and active travel. Our study also has several limitations that should be noted. The built environment measures were extracted from GIS and were missing key built environment features. More complete and precise measures of the objective built environment are needed, particularly measures of bike lanes, paths and other bike-related infrastructure, sidewalk street lighting, presence of sidewalks, marked crosswalks, and presence of public transit stops. Another limitation is that the PDI only includes pedestrian fatalities; future research should also include motor vehicle-related pedestrian injuries, which may be more prevalent. However, these data are not systematically available nationally. Thus, this study included the best available data possible at the national level. Third, the ecological fallacy may also be involved, since active travel behavior data were analyzed at the county level. Other variables, such as distance between home and work, may mediate the relationship between walkability and active travel. However, this measure is not available through the ACS. Further research is needed to explore additional variables, which cannot be ruled out by these analyses, and may affect the county-specific relationship between the built environment and active travel. Fourth, data were cross-sectional, precluding causal interpretation. Finally, there is some possible overlap between the PDI measure and the active transport outcomes. However, we examined correlations between our outcomes and the PDI, which were all below *r* = 0.50, suggesting that this should not have affected our results. Furthermore, we included biking as an outcome and found similar results with the walk to work measure. We also examined the variance inflation factor for collinearity and it was <10.

Results from this study show that county-level walkability is associated with active travel both directly and indirectly through partial mediation of an index of pedestrian safety. Results suggest that even a small increase in walkability could lead to a large change in the prevalence of people using active travel modes to get to and from work. Communities need rigorous scientific evidence to inform future policy decisions on how to increase active travel in communities. Although built environment changes require long-term planning, results of this study provide evidence that developing walkable neighborhoods is associated with increased healthy behavior and reduced pedestrian-related fatalities, which can have lasting health effects and provide one possible solution to help combat the obesity epidemic and effect positive future health behavior.

## Author Contributions

SS, the primary author of this paper, developed the research questions, designed the original data analysis, as well as drafted the paper, and had final responsibility for the decision to submit this paper for publication. LN constructed study measures and conducted the statistical analyses, and contributed to the interpretation of the analysis, results, and drafting of the paper. HZ constructed the GIS walkability and traffic calming scales and reviewed and approved the final version of this paper. JC led the original data collection effort for the larger study and contributed to the interpretation of the analysis, results, and drafting of the paper.

## Conflict of Interest Statement

The authors declare that the research was conducted in the absence of any commercial or financial relationships that could be construed as a potential conflict of interest.
